# Repurposing EGFR Inhibitors for Oral Cancer Pain and Opioid Tolerance

**DOI:** 10.3390/ph16111558

**Published:** 2023-11-03

**Authors:** Maria Daniela Santi, Morgan Zhang, Naijiang Liu, Chi T. Viet, Tongxin Xie, Dane D. Jensen, Moran Amit, Huilin Pan, Yi Ye

**Affiliations:** 1 Translational Research Center, College of Dentistry, New York University, New York, NY 10010, USA; mdaniela0505@gmail.com (M.D.S.); mz3259@nyu.edu (M.Z.); nl2782@nyu.edu (N.L.); ddj3@nyu.edu (D.D.J.); 2 Pain Research Center, Department of Molecular Pathobiology, College of Dentistry, New York University, New York, NY 10010, USA; 3 Department of Oral and Maxillofacial Surgery, School of Dentistry, Loma Linda University, Loma Linda, CA 92350, USA; cviet@llu.edu; 4 Department of Head and Neck Surgery, The University of Texas MD Anderson Cancer Center, Houston, TX 77030, USA; txxie@mdanderson.org (T.X.); moranamit@gmail.com (M.A.); 5 Center for Neuroscience and Pain Research, Department of Anesthesiology and Perioperative Medicine, University of Texas MD Anderson Cancer Center, Houston, TX 77030, USA; huilinpan@mdanderson.org

**Keywords:** oral cancer pain, EGFR, morphine tolerance, µ-opioid receptor, NMDA receptor

## Abstract

Oral cancer pain remains a significant public health concern. Despite the development of improved treatments, pain continues to be a debilitating clinical feature of the disease, leading to reduced oral mobility and diminished quality of life. Opioids are the gold standard treatment for moderate-to-severe oral cancer pain; however, chronic opioid administration leads to hyperalgesia, tolerance, and dependence. The aim of this review is to present accumulating evidence that epidermal growth factor receptor (EGFR) signaling, often dysregulated in cancer, is also an emerging signaling pathway critically involved in pain and opioid tolerance. We presented preclinical and clinical data to demonstrate how repurposing EGFR inhibitors typically used for cancer treatment could be an effective pharmacological strategy to treat oral cancer pain and to prevent or delay the development of opioid tolerance. We also propose that EGFR interaction with the µ-opioid receptor and glutamate N-methyl-D-aspartate receptor could be two novel downstream mechanisms contributing to pain and morphine tolerance. Most data presented here support that repurposing EGFR inhibitors as non-opioid analgesics in oral cancer pain is promising and warrants further research.

## 1. Introduction

Oral cancer patients tend to experience significantly more pain than other cancer sufferers [1,2,3,4]. Among these patients, pain is often rated as the worst symptom, leading to restricted oral function such as eating, drinking, and speaking, and overall poor quality of life [1,5]. Neuropathic pain, a pathological state caused by abnormalities of the somatic nervous system [6], is regarded as the least tolerable symptom of advanced oral cancer [7]. Neuropathic pain is characterized by spontaneous pain, hyperalgesia, and mechanical allodynia and is often associated with anxiety, depression, and reduced quality of life [6,8,9]. Pain can result from the tumor itself (through mass effect, ulceration, inflammation, or invasion) [3,10,11,12,13,14,15] or from the therapeutic approaches used to combat the disease, such as surgery, radiotherapy, chemotherapy, and other targeted treatments [16]. Oral cancer patients need routine assessments of their pain and functional status to be provided with the most appropriate pain management. To date, opioid analgesics remain the first choice for the management of oral cancer pain [17]. However, these agents are not without their share of pitfalls. Opioid analgesics do not always provide complete pain relief and do little to restore function, such as swallowing and oral mobility. Moreover, regular use of opioids is associated with the development of analgesic tolerance and dependence, which fuel the opioid crisis we currently find ourselves in [18]. Therefore, safer and more effective approaches for managing oral cancer pain are urgently needed. Although oral cancer pain is a complex pathological process and a formidable clinical problem, many new reports [4,7,14,19,20,21,22,23,24,25,26] indicate promising ways to improve pain management. One strategy is inhibiting the epidermal growth factor receptor (EGFR) activity. In this review, we explore current clinical and preclinical evidence that supports the potential use of EGFR inhibitors to address pain and morphine tolerance in oral cancer, in addition to their established role as effective anticancer agents. The data summarized here were retrieved from PubMed with keywords search for EGFR + pain, EGFR + morphine tolerance, EGFR + oral cancer, MOR+ morphine tolerance, NMDAR + pain, NMDAR + MOR, and NMDAR + EGFR. 

## 2. EGFR and Its Known Signaling Pathways

EGFR (ErbB1, HER1) is a 170 KD transmembrane protein that belongs to the receptor tyrosine kinase (RTK) family and the ErbB/HER family [27,28]. EGFR plays essential roles in regulating prenatal development, adult tissue homeostasis, cell growth, proliferation, migration, metabolism, differentiation, and survival [8,29]. In physiological conditions, EGFR is expressed in human skin, placenta, endocrine tissues, immune cells, and the central nervous system [30,31]. EGFR is most well known for its involvement in tumorigenesis and cancer progression [8,32,33]. Notably, EGFR is highly expressed in the rat, mouse, and human dorsal root ganglia (DRG) [34,35]. EGFR is also expressed in the spinal cord of humans and mice [36], as well as rats [37].

Structurally, EGFR is a single-chain glycoprotein comprising an ectodomain, a transmembrane domain, and a cytoplasmic domain that is composed of two subunits: the tyrosine kinase domain and the tyrosine C-terminal [29]. The ectodomain binds soluble ligands leading to conformational alterations that activate the receptor by homo- or heterodimerization with other HER receptors or other RTKs, such as hepatocyte growth factor receptor (HGFR or MET) and the insulin-like growth factor 1 (IGF-1). This, in turn, activates the intracellular kinase domain of EGFR, which results in autophosphorylation of the tyrosine residues at the C-terminal [29]. EGFR has seven reported ligands, each having a different affinity for the receptor: epidermal growth factor (EGF), betacellulin (BTC), heparin-binding EGF-like growth factor (HB-EGF), and transforming growth factor alpha (TGFα) as ligands with high binding affinity, and amphiregulin (AREG), epiregulin (EREG), and epithelial mitogen (EPGN) having low binding affinity [38].

In addition, EGFR can be indirectly stimulated by the activation of G protein-coupled receptors (GPCRs). It has been reported that ligands of GPCRs, such as prostaglandin (PGE2) and gastrin-releasing peptide (GRP), which are often overexpressed in oral cancer, can activate GPCR and Src-mediated matrix metalloproteinase (MMP), leading to the cleavage and release of EGFR ligands [39]. The subsequent activation of EGFR can promote the expression of COX2 and PGE2, giving rise to a self-perpetuating positive feedback loop [39].

**Figure 1:**
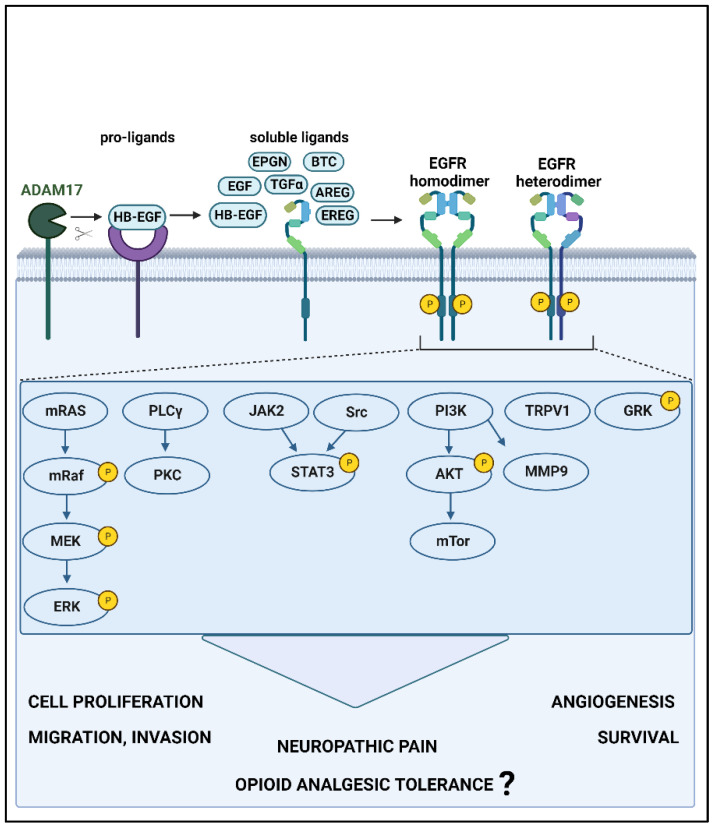
EGFR signaling pathways and downstream effects. When EGFR interacts with high- or low-affinity ligands, resultant conformational changes lead to homo- or heterodimerization and subsequent activation by phosphorylation. This stimulates one or more signaling pathways, which lead to carcinogenesis and pain and may induce opioid tolerance. Furthermore, EGFR leads to the activation of receptors (TRPV1: transient receptor potential cation channel subfamily V member 1) and kinases (GRK: G protein-coupled receptor kinase 2) that are involved in pain and opioid receptor regulation. ADAM17: ADAM metallopeptidase domain 17; EGF: epidermal growth factor, BTC: betacellulin, HB-EGF: heparin-binding EGF-like growth factor, TGFα: transforming growth factor alpha, AREG: amphiregulin, EREG: epiregulin, EPGN: epithelial mitogen, EGFR: epidermal growth factor receptor, MEK: mitogen-activated protein kinase, ERK: extracellular signal-regulated kinase, PLCγ: phospholipase C gamma, JAK2: Janus kinase 2, Src: Src-family kinase, STAT3: signal transducer and activator of transcription 3, PI3K: phosphoinositide 3-kinase, mTORC: mammalian target of rapamycin complex, MMP9: matrix metalloproteinase-9. P: phosphorylation. This figure was generated using Biorender.com.

The interaction between EGFR and its ligands can activate a number of different signaling pathways, such as MAPK, PLCγ/PKC, PI3K/AKT/mTOR, and the JAK/STAT pathway [8,39] (Figure 1). While the development and progression of oral squamous cell carcinoma (OSCC) are known to be driven via EGFR-HB-EGF and EGFR-EREG interactions [40,41,42] in OSCC cells, pain is associated with EREG- or HB-EGF-mediated activation of EGFR [19,43,44]. Furthermore, morphine tolerance and dependence may cause downregulation of µ-opioid receptors via EGFR-EGF in rats and HEK293 cells [25,45] and EGFR-EREG in mice [43].

## 3. EGFR in Oral Cancer Treatment in Clinical Studies 

Head and neck squamous cell carcinoma (HNSCC) is the sixth most common cancer ranked by incidence and mortality globally, with most lesions in the oral cavityOSCC comprises more than 90% of all oral malignancies [46]. Even with rigrous treatment, recurrence, and metastasis are common, accounting for a poor survival rate of 20–40% [47].

In 1991, Santini et al. described EGFR as a biomarker for HNSCC [48] after it was found to be overexpressed in human tumor tissues [49,50,51,52]. In response to this finding, EGFR-targeted therapy arose as an alternative therapeutic option for HNSCC. To date, there are two fundamental EGFR-targeted approaches for cancer treatment. One approach involves the use of monoclonal antibodies (mAbs) that bind to the extracellular domain of EGFR, preventing receptor dimerization and subsequent activation, thereby leading to EGFR downregulation. Cetuximab is the only mAb approved by the Food and Drug Administration (FDA) and European Medicines Agency (EMA) for the treatment of locally advanced HNSCC in combination with radiotherapy and as monotherapy for metastatic cases [53]. Despite panitumumab having failed to show efficacy in HNSCC (NCT00460265, NCT00820248), other mAbs, such as zalutumumab and nimotuzumab, currently in phase III clinical trials, have demonstrated promising results (NCT00382031, NCT00957086). Skin rashes, a main side effect of these treatments, are considered an indicator of drug efficacy [53,54,55].

The second approach involves the use of small molecules as irreversible tyrosine kinase inhibitors (TKIs). TKIs bind to the EGFR-intracellular tyrosine kinase domain and inhibit EGFR phosphorylation and subsequent downstream signaling. At present, there are no TKIs that are FDA-approved for HNSCC treatment. Gefitinib, a TKI currently used for lung cancer, failed to improve the overall survival and the progression-free survival in HNSCC as either monotherapy or in combination with methotrexate (NCT00206219) or docetaxel (NCT00088907). On the other hand, erlotinib, approved for use in pancreatic cancer, has shown promising results in HNSCC clinical trials. Erlotinib used alone, in combination with cisplatin, or as an adjuvant improved overall survival, decreased HNSCC proliferation (NCT01515137), and prevented recurrence [56]. The non-selective EGFR/HER2 inhibitor, lapatinib, approved for use in breast cancer, did not provide a survival benefit in HNSCC (NCT00098631, NCT01044433, and NCT00424255), either alone or in combination with chemoradiotherapy. Vandetanib, an EGFR/VGFR2 inhibitor, is currently being studied in HNSCC preclinical models and has shown promising results [53].

The current challenges of the HNSCC treatment with EGFR TKIs or mAbs lie in identifying possible EGFR mutations and counteracting the possible resistance mechanisms that these mutations produce. Although resistance to cetuximab has not been a concern in HNSCC, several resistance mechanisms have been reported in other cancer types, such as colorectal cancer and non-small cell lung cancer. Overexpression of EGFR ligands, nuclear translocation of EGFR, KRAS mutation, and PTEN loss are examples of resistance mechanisms to anti-EGFR mAbs. This resistance leads to constant activation of EGFR and PI3K/AKT signaling. Some of these resistance mechanisms also apply to TKIs, along with evidence of EGFR mutations, IGF-1R activation, and histologic transformation [39].

Recent evidence suggests that the use of EGFR inhibitors in a multitargeted approach can be more beneficial for the treatment of HNSCC. Several studies have demonstrated that the combination of more than one HER inhibitors (known as horizontal targeting) or the combination of HER inhibitors with other TKIs (known as vertical targeting) improves treatment efficacy and prevents resistance [39,57]. The issue of resistance to EGFR inhibitors could also be addressed by targeting cancer metabolism, as evidenced by recent studies reporting a link between lipid metabolism and activation of downstream EGFR pathways, such as AKT [39,58,59]. Li et al. reported a link between cancer metabolism and cetuximab resistance in HNSCC in 2015 [60].

## 4. EGFR in Pain and Morphine Tolerance 

### 4.1. Clinical Studies and Genetic Associations 

The earliest evidence that TKIs might alleviate chronic pain came from clinical observations in non-small cell lung cancer (NSCLC) patients receiving erlotinib treatment. These patients not only lived longer but also had improved tumor-related pain, physical function, and overall quality of life [8,61,62]. Afatinib, another TKI, was also reported to decrease pain in advanced lung cancer patients [63]. Moreover, a case report showed that afatinib was effective in relieving pain in a patient with severe neuropathic pain [64]. In addition, a phase III trial in patients with advanced SCC of the lung after having received first-line platinum-based chemotherapy demonstrated that afatinib improved progression-free survival, overall survival, and decreased pain [65].

Regarding mAbs, rectal cancer patients reported pain relief after treatment with cetuximab despite tumor progression [66]. Pain relief was also reported among neuropathic pain patients after treatment with cetuximab or panitumumab [67,68]. This was echoed in another study by Kersten and colleagues, who showed pain relief in 20 neuropathic pain patients of both sexes undergoing treatment with cetuximab or panitumumab, reporting skin reactions as the most common side effect [67]. The same researchers also reported a similar pain reduction response for treatment with TKIs erlotinib and gefitinib [9,69]. 

Genetic links that involve EGFR and pain were first reported in temporomandibular disorders, a common condition of orofacial pain by Dr. Luda Diatchenko’s group. In the Orofacial Pain: Prospective Evaluation and Risk Assessment (OPPERA) project, it was shown that the single nucleotide polymorphisms (SNPs) of EGFR and one of its cognate ligands (EREG) are associated with the development of chronic pain in temporomandibular disorders [43], especially in females of mixed European descendants. In addition, EREG SNPs, which lead to decreased circulating EREG mRNA, were negatively correlated with TMD development. Similar results were reported by Verma et al., who performed a systematically screen of SNPs in all gene loci belonging to EGFR and its ligands in the OPPERA cohort and found that *EREG* SNPs were associated with chronic pain intensity [70]. However, after characterization of the same *EREG* SNPs and acute and chronic pain states in OPPERA, they paradoxically found that the same EREG SNPs variant that was protective for chronic pain increased the risk for acute pain intensity. Using an independent cohort of TMD patients from the U.K. Biobank (UKB), the authors further validated the dichotomous role of EREG in acute and chronic pain states [70].

Genetic links that involved EGFR and pain were also found in other painful disease conditions. Recently, it was demonstrated that there was a positive association between tumors with EGFR mutations and higher rates of pain in response to palliative radiotherapy in an analysis of NSCLC patient-reported outcomes [71], further supporting the involvement of EGFR in both cancer progression and pain signaling. In an interactome study aimed to identify ligand-receptor pathways relevant to pain in a cell type-specific manner, Dr. Price’s group created interactome maps between human DRG sensory neurons and rheumatoid arthritis-associated synovial macrophages and pancreatic tumor tissue using published RNA-seq datasets. They found the abundance of EGFR ligands in the periphery and its receptor expressed on DRGs are important for persistent pain associated with these two painful disease states [44]. In a recent genome-wide association study (GWAS) performed on 23,000 participants with musculoskeletal pain from the UKB, the authors identified a hit in SNP (rs549224715) on chromosome 4 to be significantly associated with analgesic ladder switch from non-steroidal anti-inflammatory drugs (NSAIDs) to opioids for pain management by comparing NSAID users and opioids users. In the subsequent network and pathway analysis on functional genes located on the significant loci, EGFR was identified as a central hub [72], further suggesting its potential role in pain progression.

### 4.2. EGFR Involvement in Pain in Animal Models 

In most animal studies, EGFR signaling activation was pronociceptive (summarized in Table 1), where EGFR ligands induced or aggravated mechanical allodynia, thermal hypersensitivity, cold hypersensitivity, or other nociceptive behaviors. In the initial report on EGFR involvement in pain, it was shown that EREG is the only ligand among those tested (e.g., amphiregulin, EGF, TGF-α, Betacellulin) that can not only elicit an acute nociceptive response but also amplify existing pain when injected intrathecally [70]. Recently, it has been shown that other ligands like EGF [25], HB-EGF, and a peptide toxin mimicking the EGF domain of HB-EGF [69,72] can also elicit an acute nociceptive response when injected at the periphery. Downregulation of TGFαin DRGs, has been shown to suppress CCL2/CCR2 signaling and reduce pain in a surgically induced osteoarthritis pain model [73]. In addition, although directly behavioral data are lacking on epigen and pain, transgenic mice overexpressing epigen exhibit signs of neuropathies such as nerve demyelination, axon degeneration, and muscular atrophy in an EGFR-dependent manner [74]. 

Accordingly, inhibition of EGFR signaling reduces certain nociceptive behaviors (Table 1). EGFR signaling can be inhibited by a reduction in EGFR ligand expression [75,76,77] or release [73], by scavenging of EGFR ligands [25], or using EGFR TKIs (e.g., gefitinib, lapatinib, AG1478) [39]. 

It is important to note that the involvement of EGFR signaling in pain is context dependent, and its activation may even be neuroprotective or analgesic. Ligands like EREG have a dicrotous role in pain. EREG was discovered to be analgesic during acute pain [67] while being pronociceptive for chronic pain [39]. TGFα and HB-EGF were found to be elevated in degenerated osteoarthritis cartilage [78]. Accordingly, it was proposed that EGFR inhibitors could be repurposed for joint pain treatment, which was supported by several rodent studies with injury-induced osteoarthritis [75,76,79]. However, EGFR activation by intra-articular administration of TGFα, rather than EGFR inhibition, was reported to attenuate osteoarthritis pain [74,75,79]. In a rat visceral pain model, increased EGF levels have been implicated in upregulating serotonin transporter-mediated serotonin uptake in intestinal epithelial cells, therefore lowering the serotonin level and ultimately reducing rather than producing visceral hypersensitivity [76]. Finally, in a spinal nerve ligation model, where EGFR ligands are shown to play a role in the initiation of nerve injury-induced allodynia, but as the disease condition progresses, multiple additional effectors sustain the pain, so EGFR inhibition alone cannot reverse nociceptive responses [25]. 

**Table 1:** EGFR involvement in pain in animal models.

Category	Procedure or Animal Models	Species	EGFR Activation		EGFR Inhibition		Ref.
			Induced by	Behavioral Outcomes	Induced by	Behavioral Outcomes	
Nociceptive	None	Mouse	HB-EGF injected into the paw	Mechanical allodynia			[44]
		Mouse	HB-EGF mimicking toxin (intraplanar)	Mechanical allodynia, thermal hypersensitivity			[80]
		Rat	EGF (i.t.)	Mechanical allodynia			[25]
		Mouse	Epiregulin (i.t.)	Mechanical allodynia, thermal hypersensitivity			[43]
	ADAM17 hypomorphic mutant	Mouse			Reduced release of EGFR ligands	Reduced mechanical allodynia, heat hypersensitivity, and cold hypersensitivity	[81]
Inflammatory	Intraplantar Injection of formalin	Mouse	Epiregulin (i.t.)	Analgesic during the early phase; Aggravated late-phase nociceptive behavior (lick/bite)			[43]
		Mouse	EGF, amphiregulin betacellulin TGFα (i.t.)	No effect			
		Mouse			AG 1478, gefitinib, lapatinib (i.p.)	Reversal of late-phase nociceptive behavior (licking/biting)	
	Injection of Complete Freund’s adjuvant	Mouse			AG 1478, gefitinib, lapatinib (i.p.)	Reversal of mechanical allodynia	[43]
	Injection of carrageenan	Mouse			AG 1478, gefitinib, lapatinib (i.p.)	Reversal of thermal hypersensitivity	[43]
	Anterior cruciate ligament, transection, and partial medial meniscectomy	Rat			AG1478 (infusion)	Reduced osteoarthritis at 4 and 7, not 10 wk postsurgery in males	[75,82]
	DMM-induced osteoarthritis	Mouse			Downregulation of TGFα in DRGs by miR-183	Reduced mechanical allodynia at 8 wk postsurgery in males	[73]
	DMM-induced osteoarthritis	Mouse	HB-EGF overexpression, or TGFα (intra-articular)	Reversal of mechanical allodynia after 1 week postsurgery	Gefitinib (oral)	No reversal of mechanical allodynia after 1 wk postsurgery	[78]
	DMM-induced osteoarthritis in EGFR knockout mice	Mouse			Reduced intra-articular EGFR expression	Development of mechanical allodynia 1 month postsurgery	[83]
	Tibial loading of 6 Newton	Mouse	Intra-articular HB-EGF overexpression	Reversal of mechanical allodynia			[77]
	Intra-colonic infusion of acetic acid	Rat			Lower EGF levels in plasma and colon	Development of visceral hypersensitivity	[84]
Neuropathic	Spared nerve injury	Mouse			EGFR inhibitor III (i.p.)	Reversal of mechanical allodynia	[85]
		Mouse			AG 1478, gefitinib, lapatinib (i.p.)	Reversal of mechanical allodynia	[43]
	Chronic constriction injury	Mouse			AG 1478, gefitinib, lapatinib (i.p.)	Reversal of mechanical allodynia	[43]
		Rat			Erlotinib (i.p.)	Reversal of mechanical allodynia, thermal hypersensitivity, cold hypersensitivity	[86]
					Gefitinib, AG 1478, falnidamol, EGFRi 324674 (i.p.)	Reversal of mechanical allodynia	
					Lapatinib, afatinib (i.p.)	Reversal of mechanical allodynia	
					Geniposide (i.p.)	Reversal of mechanical allodynia, thermal hypersensitivity	[87]
	Lumbar spinal nerve ligation	Rat			Imatinib, gefitinib, EGF-scavenging molecule (i.t.)	No reversal of mechanical allodynia	[25]
	Injection of oxaliplatin	Mouse			Erlotinib, gefitinib, AG 1478 (i.p.)	Reversal of mechanical allodynia	[86]
Mixed	Injecting cancer cell supernatant into the tongue	Mouse			Cetuximab (i.p.)	Reversal of orofacial nociceptive behavior	[19]
	Chronic DRG compression	Rat			Gefitinib, EGFR siRNA (i.t.)	Reversal of mechanical allodynia, thermal hypersensitivity, cold hypersensitivity	[88]

**Note:** EGFR signaling activation was nociceptive in all listed models except the models depicted in italics. The following abbreviations have been used: DMM: destabilization of the medial meniscus by surgery; DRG: dorsal root ganglions; i.t.: intrathecal; i.p.: intraperitoneal; wk: weeks.

### 4.3. Downstream Signaling Cascade of EGFR Signaling in Pain

The downstream effects of EGFR are mediated by one of several important signaling pathways (listed below) to induce hypersensitivity by EGFR activation or reduce pain by EGFR inhibition.

(1) PI3K/AKT/mTOR (phosphatidylinositol 3-kinase/protein kinase B/mammalian target of rapamycin): A study of nocifensive behaviors in a formalin-induced inflammatory pain mouse model [43] revealed that EREG upregulation in the blood might activate EGFRs on DRG neurons through the mTOR signaling pathway, which increases phosphorylation of 4E-BP1 and then MMP-9 translation. MMP-9 is important in inducing early-phase neuropathic pain by activating IL-1β, TNF-α, and microglia [89]. In addition, gene expression analysis of multiple human cancer cell lines showed that the PI3K/AKT signaling pathway (including EGFR and mTOR) contained the highest number of differentially expressed genes with the nociceptive trait matched to that obtained in a mouse model of acute oral cancer pain [19];

(2) PI3K/AKT/LRP1 (phosphatidylinositol 3-kinase/protein kinase B/lipoprotein receptor-related protein 1): A study in a neuropathic pain model on rats identified that the increased excitability and excessive firing that are likely to underlie pain hypersensitivity may be caused by increased EGFR and AKT recruitment of Nav1.9, Nav1.8, and Cav1.2 by LRP1 (as the vesicular chaperone) to the apical plasma membrane and proximal stem axon of primary afferent nociceptive neurons after nerve injury [86];

(3) GCH1/BH4 (GTP cyclohydrolase-1/6R-L-erythro-5, 6, 7, 8-tetrahydrobiopterin): In rat and human DRG neurons, BH4 was proposed to bind with nNOS, resulting in increased production of NO, which sensitizes the transient receptor potential vanilloid (TRPV1) or the transient receptor potential cation channel subfamily A member 1 (TRPA1) channels [90]. A study with a neuropathic pain mouse model [85] uncovered that EGFR/Kirsten ras sarcoma virus (KRAS) signaling triggers increased GCH1 expression, leading to an increase in BH4 and persistent pain sensitivity;

(4) In sensory neurons, EGFR also affects other targets, e.g., δ-opioid receptors [91], beta-adrenergic receptors [92], calcium channels [44], cannabinoid type 1, and TRPV1 receptors [43,93], which are important for pain processing;

(5) In glial cells, e.g., satellite glial cells, EGFR is also expressed [34]. Satellite glial cells are activated by compressed DRGs and release proinflammatory cytokines, such as interleukin (IL)-1, IL-6, and TNF-α, to further activate glia; EGFR inhibition may reduce the release of proinflammatory cytokines and then relieve chronic DRG compression-induced pain hypersensitivities [34];

(6) In osteoarthritis pain, EGFR signaling activation has a tissue-dependent effect. In mouse articular cartilage, EGFR signaling may have a protective role against osteoarthritis pain by maintaining the number and properties of superficial chondrocytes, promoting chondrogenic proteoglycan 4 (Prg4) expression, and stimulating the lubrication function of the cartilage surface, which is otherwise diminished in osteoarthritis [77,78,83]. Nonetheless, in mouse DRG, TGFα activation of EGFR was reported to promote movement-provoked pain via a TGFα-mediated C-C motif chemokine ligand 2 (CCL2)/CC-chemokine receptor 2 (CCR2) signaling axis [73,75,82].

### 4.4. EGFR Involvement in Morphine Tolerance in Animal Models

EGFR has also been demonstrated to mediate opioid tolerance in several rodent models, including two chronic pain models of spinal nerve ligation (SNL)-induced peripheral neuropathies and cancer-induced bone pain (CIBP) [25,94] and pre-tolerance in morphine-naive rats [25] (summarized in Table 2) where enhanced EGFR signaling can aggravate while suppression of EGFR signaling can alleviate morphine tolerance [25,94].

EGFR signaling involved in morphine tolerance occurs predominantly in the spinal cord [25,94], including both neurons and microglia. In a CIBP model in rats, Yang et al. demonstrated that morphine tolerance correlates with a sustained increase in the protein levels of EGFR (both in microglia and neurons), p-EGFR, ERK1/2, and p-ERK1/2 in the spinal cord as well as microglia proliferation. In contrast, inhibition of EGFR signaling by intrathecal administration of AG1478 markedly attenuated the degree of morphine tolerance in morphine-treated sham or CIBP rats, as well as decreased the number of microglia, and the protein levels of EGFR, p-EGFR, ERK1/2, and p-ERK1/2 in the spinal cord [26]. In another in vivo model of opioid-induced tolerance, whereby rats were administered morphine for five consecutive days, it was demonstrated that EGFR is both necessary and sufficient to induce opioid tolerance and mechanical sensitization. Furthermore, inhibition of EGFR signaling by gefitinib restored morphine analgesic effect against mechanical allodynia, and chronic injections of EGF caused a decrease in paw withdrawal threshold, contributing to the development of pre-tolerance [25].

### 4.5. Possible Downstream Signaling Cascades of EGFR in Opioid Tolerance

#### 4.5.1. EGFR and MOR Interactions

Opioid analgesia is mediated mainly by the µ-opioid receptor (MOR), which is expressed both in the CNS (spinal cord and brain) and in the PNS (DRGs and peripheral nerves). MOR is a G protein-coupled receptor that pairs with inhibitory G proteins (Gi and Go) [95]. After acute administration, MOR agonists inhibit voltage-gated calcium channels in primary sensory neurons [96,97], resulting in an inhibition of inflammatory and pain signaling pathways. However, other mechanisms are activated when morphine is administered chronically [45,98]. Tolerance is a consequence of adaptive mechanisms functioning at different levels (cellular, synaptic, and network), in which the activity of MOR is altered in order to restore normal function following the perturbation produced by opioid agonists [98]. The following summarizes the proposed mechanisms underlie opioid tolerance:

(1) Receptor tolerance: loss of surface MOR receptors, phosphorylation of MOR, internalization/endocytosis, sequestration/recycling, and downregulation/desensitization [99];
(2) Cellular tolerance and withdrawal: upregulation of cAMP, increase in adenylyl cyclase (AC) activity and sensitization [45];
(3) Synaptic plasticity in tolerance and hyperalgesia: potentiated presynaptic NMDAR activity at the spinal cord level [100,101].

**Figure 2:**
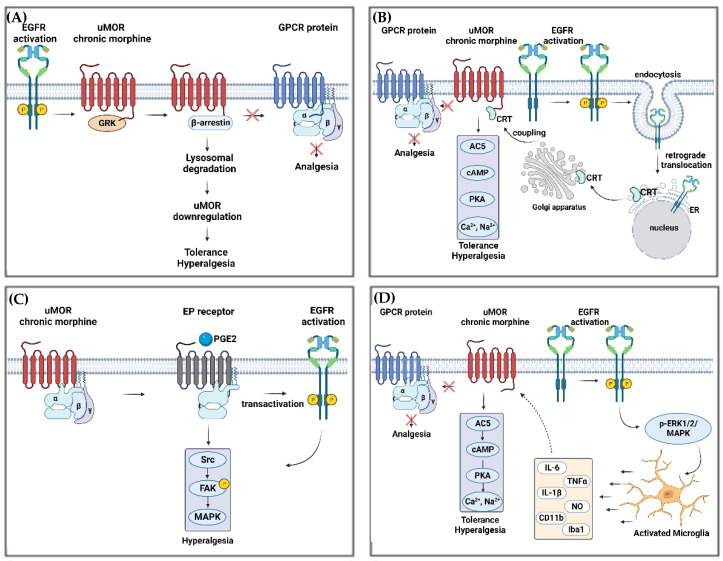
Pathways of morphine tolerance potentially regulated by EGFR and MOR interactions. (**A**) EGFR activation due to its phosphorylation (P), generates pre-tolerance by interacting with MOR-activated by acute morphine administration. This leads to MOR lysosomal degradation via β-arrestin association, a subsequent impediment to interact with GPCR (comprised by α, β and γ subunits), and finally, hyperalgesia and tolerance. (**B**) Chronic morphine administration blocks the interaction between MOR and GPCR and activates EGFR. Subsequently, EGFR undergoes retrograde translocation to the ER, resulting in the trafficking of CRT (Calreticulin) to the Golgi before migrating again to the membrane and finally couples to MOR. This coupling leads to the activation of the AC5/cAMP/PKA signaling cascade, generating the release of higher concentrations of Ca^2+^ and Na^+^ within synaptoneurosome, which contribute to the development of tolerance and hyperalgesia. (**C**) MOR’s chronic stimulation by morphine leads to PGE2 activation of EP receptor, which, together with EGFR’s transactivation, activates Src/FAK/MAPK signaling, a pathway well known for hyperalgesia. (**D**) EGFR activation triggers the p-ERK1/2/MAPK pathway, which activates microglia at the CNS. Activated microglia release cytokines that act on MOR chronic morphine-stimulated, leading to the activation of AC5/cAMP/PKA signaling cascade and the release of Ca^2+^ and Na^+^. The following abbreviations have been used: EGFR: epidermal growth factor receptor; GPCR: G protein-coupled receptors; ER: endoplasmic reticulum; ERK: extracellular signal-regulated kinase; CRT: calreticulin; AC5: adenylyl cyclase 5; cAMP: adenosine monophosphate; PKA: protein kinase A; PGE2: prostaglandin E2; EP: prostaglandin E receptor; Src: Src-family kinase; FAK: focal adhesion kinase; MAPK: mitogen-activated protein kinase; p-ERK1/2: extracellular signal-related kinase. Own figure created using BioRender.com.

The cross-talk between MOR and EGFR was explored in vitro in primary cultured cortical neurons (from the whole cortex) isolated from neonatal (postnatal days 1–2) by Zhao et al. They found that, after chronic morphine treatment, adaptive changes in both MOR and EGFR signal systems lead to an AC5 superactivation and subsequent development of tolerance. Furthermore, in N_2_A-EGFR cells (a neuroblastoma cell line expressing EGFR), the same group observed that chronic morphine administration led to increased EGFR phosphorylation and translocation to the endoplasmic reticulum, which was essential for posterior MOR-CRT (calreticulin) tethering, leading to increased AC5 activity [45]. Studies of primary afferent nociceptors in mice showed that EGFR is involved in MOR prolongation of hyperalgesia induced by PGE_2_ [8,102,103]. Moreover, it has been shown that in A431 cells (epidermoid carcinoma cells that express EGFR) as well as HEK293 cells co-expressing GPCR kinase-2 and EGFR, EGFR activation by EGF led to a downregulation of the opioid receptor as a consequence of its interaction with G proteins, which activated GPCR kinases [91,104]. It was reported that, after activation, MOR associates with ꞵ-arrestins, thereby inducing receptor internalization and downregulation in different in vivo models [8,102,105]. Figure 2 summarizes pathways of morphine tolerance potentially regulated by EGFR and MOR interactions.

#### 4.5.2. EGFR and NMDA Receptor Interactions

Glutamate N-methyl-D-aspartate receptors (NMDAR) are heterothermies consisting of two obligatory subunits (GluN1) and two regulatory subunits (GluN2A-2D or GluN3), with GluN2A and GluN2B being the most extensively studied due to their role in synaptic activity [106]. NMDAR plays a role in physiological processes within the CNS (synaptogenesis, plasticity) and in various neurological disorders, such as schizophrenia, epilepsy, ischemic brain damage, and neurodegenerative disorders [106,107]. Furthermore, increased activity of spinal NMDAR, particularly α2δ-1-bound NMDAR [108,109,110], has a major role in the development of central sensitization and neuropathic pain [111]. In the hippocampus, EGF treatment increases NMDAR phosphorylation and surface expression of the GluN2B, contributing to long-term potentiation (LTP) [106].

NMDAR expressed in primary sensory neurons, and their central terminals in the spinal cord also promotes opioid-induced hyperalgesia and analgesic tolerance. Increased glutamate activity under chronic morphine can induce hyperalgesia/allodynia and consequently counteract opioid analgesia. That event occurs at different levels:

(1) Neuronal circuits (synaptic signaling), such as suppressing MOR signaling via (a) dimerization with presynaptic mGluR5 to potentiate NMDAR synaptic expression and activity, and (b) complex with three functionally interrelated MAPKs (ERK1/2, p38, and JNK) to induce a tonic activation of presynaptic NMDARs [112] at primary afferent central terminals;

(2) Cellular adaptations, which include gene expression [113,114,115] and the reduction in MOR function. An exemplar study of the latter is that increased activity of NMDAR by PKC/PKA-induced phosphorylation of its GluN2A and GluN2B subunits leads to higher calcium influx, increased release of NO, and subsequent negative regulation of MOR activity [116].

Pharmacological studies have identified several signaling proteins involved in the interaction between glutamate/NMDAR and opioid/MOR tolerance. Some of them include nitric oxide synthase (NOS), protein kinase C (PKC), protein kinase A (PKA), calcium (Ca^2+^)/calmodulin (CaM)-dependent kinase II (CaMKII), delta-opioid receptor (DOR) and the regulators of G-protein signaling (RGS) proteins [114,117,118].

**Figure 3:**
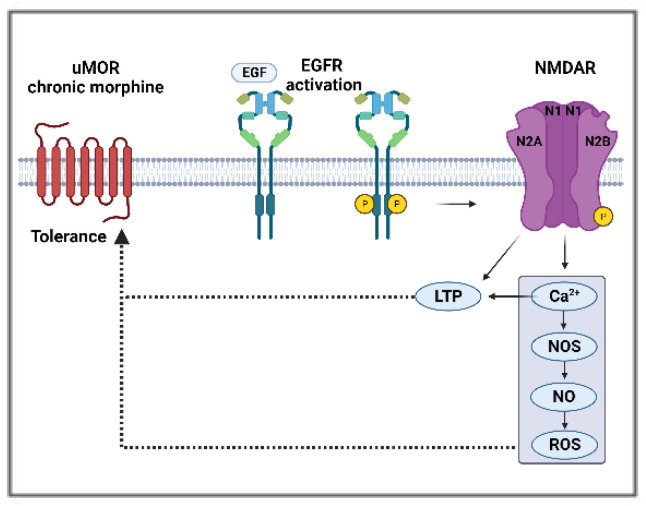
Potential mechanism of morphine tolerance regulated by EGFR and NMDAR interactions (P:phosphorylation).

EGFR signaling was shown to be responsible for an increase in NMDAR calcium currents as a consequence of in vitro EGF-mediated increased expression and phosphorylation of GluN2B subunit in primary hippocampal cells cultured from Sprague–Dawley rat fetuses on embryonic days 17–19 [106]. The same subunit was reported as a substrate for EGFR in glioma cells [119], where EGFR phosphorylates the COOH-terminal domain of the subunit, leading to an increase in glutamate-NMDAR signaling. Similarly, in an HB-EGF knockout mice model, the absence of the EGFR ligand led to reduced protein levels of the GluN1 subunit and calcium influx [120]. LTP, a maladaptive plasticity involved in numerous pathologies, including hyperalgesia, drug addiction, and tolerance, can be triggered by opioids in the spinal cord of rats and mice [100,101]. EGFR signaling activation by EGF enhances LTP in hippocampal primary cultures derived from rat fetuses [106]. EGFR activation may lead to synaptic NMDAR hypersensitivity in the pain pathways to induce morphine tolerance (Figure 3).

## 5. Challenges

To implement EGFR inhibitors for cancer pain control, several challenges remain. The underlying mechanisms of EGFR’s pronociceptive actions are still evolving. The potential interaction of EGFR with MOR and NMDAR reviewed above has not been studied directly in the pain pathways. Resistance to EGFR inhibitors may potentially limit their prolonged use for pain management. Lastly, the content-dependent action of EGFR ligands in pain and the paradox of EGFR activation suppressing acute pain in certain conditions warrant a closer examination of the nuanced roles of each ligand and their downstream signaling pathways. Nevertheless, similar strategies used in treating cancer can be adopted for pain, such as developing new generations of EGFR inhibitors, employing different combinations of EGFR inhibitors, and/or combinations with non-EGFR inhibitors to develop non-opioid analgesics to treat oral cancer pain.

## 6. Conclusions and Perspectives

EGFR inhibitors for cancer therapies are an area of active research, with many already on the market [121,122,123,124,125,126,127,128,129,130,131,132,133]. Emerging evidence suggests that EGFR expressed along the pain signaling pathway may play a significant role in regulating pain and promoting opioid analgesic tolerance and dependence. EGFR inhibitors could be repurposed for oral cancer pain management because their safety profile is well known. The benefits of repurposing EGFR inhibitors as non-opioid analgesics are promising and clearly warrant further research. This is particularly crucial in our current climate as we endeavor to fight the opioid overdose epidemic.

## Figures and Tables

**Table 2:** EGFR involvement in morphine tolerance and pre-tolerance in animal models.

Animal Models		EGFR Activation		EGFR Inhibition		Ref.
		Induced by	Behavioral Outcomes	Induced by	Behavioral Outcomes	
i.t. morphine	Rat	EGF (4 days)	Production of pre-tolerance and thermal hypersensitivity			[25]
Lumbar spinal nerve ligation Morphine (i.t. or subcutaneous)	Rat			Gefitinib (i.t. or subcutaneous)	Reversal of tolerance mechanical allodynia and thermal hypersensitivity	[25]
				EGF-scavenging molecule (i.t.)	Reversal of tolerance and mechanical allodynia	
Injecting Walker 256 mammary gland carcinoma cells into tibias + morphine (i.t.)	Rat			AG 1478 (i.t.)	Reversal of tolerance and mechanical allodynia	[94]

**Note:** i.t.: intrathecal.

## Data Availability

Data are contained within the article.
